# Effectiveness of a peer-led HIV prevention intervention in secondary schools in Rwanda: results from a non-randomized controlled trial

**DOI:** 10.1186/1471-2458-12-729

**Published:** 2012-09-01

**Authors:** Kristien Michielsen, Roxanne Beauclair, Wim Delva, Kristien Roelens, Ronan Van Rossem, Marleen Temmerman

**Affiliations:** 1 International Centre for Reproductive Health, Faculty of Medicine and Health Sciences, Ghent University, De Pintelaan, 185 6K4, 9000, Gent, Belgium; 2 South African Centre for Epidemiological Modelling and Analysis, Stellenbosch Institute for Advanced Study (STIAS), Stellenbosch, South Africa; 3 Department of Obstetrics and Gynaecology, Faculty of Medicine and Health Sciences, Ghent University, De Pintelaan, 185P3, 9000, Gent, Belgium; 4 Department of Sociology, Faculty of Political and Social Sciences, Ghent University, Korte Meer 3, 9000, Gent, Belgium

**Keywords:** Peer education, HIV prevention, Effectiveness, Young people, Rwanda

## Abstract

**Background:**

While the HIV epidemic is levelling off in sub-Saharan Africa, it remains at an unacceptably high level. Young people aged 15-24 years remain particularly vulnerable, resulting in a regional HIV prevalence of 1.4% in young men and 3.3% in young women. This study assesses the effectiveness of a peer-led HIV prevention intervention in secondary schools in Rwanda on young people’s sexual behavior, HIV knowledge and attitudes.

**Methods:**

In a non-randomized longitudinal controlled trial, fourteen schools were selected in two neighboring districts in Rwanda Bugesera (intervention) and Rwamagana (control). Students (n = 1950) in eight intervention and six control schools participated in three surveys (baseline, six and twelve months in the intervention). Analysis was done using linear and logistic regression using generalized estimation equations adjusted for propensity score.

**Results:**

The overall retention rate was 72%. Time trends in sexual risk behavior (being sexually active, sex in last six months, condom use at last sex) were not significantly different in students from intervention and control schools, nor was the intervention associated with increased knowledge, perceived severity or perceived susceptibility. It did significantly reduce reported stigma.

**Conclusions:**

Analyzing this and other interventions, we identified several reasons for the observed limited effectiveness of peer education: 1) intervention activities (spreading information) are not tuned to objectives (changing behavior); 2) young people prefer receiving HIV information from other sources than peers; 3) outcome indicators are not adequate and the context of the relationship in which sex occurs and the context in which sex occurs is ignored. Effectiveness of peer education may increase through integration in holistic interventions and redefining peer educators’ role as focal points for sensitization and referral to experts and services. Finally, we argue that a narrow focus on sexual risks will never significantly turn the tide.

## Background

With 22.9 million people living with HIV and 1.9 million new infections in 2010 the HIV epidemic seems to be levelling off in sub-Saharan Africa, but remains at an unacceptably high level. Nearly half of the new HIV infections occur among young people aged 15-24 years, resulting in a regional HIV prevalence of 1.4% in young males and 3.3% in young females [1]. Hence, changing sexual behavior in this group is crucial in tackling the pandemic [2]. Since no vaccine or cure is available, the focus should be on behavioral prevention such as, delaying the onset of sex, reducing the number of sex partners and increasing condom use. Despite many efforts and international engagements, youth still lack the knowledge, tools and support they need to practice these HIV risk-reduction strategies [3,4].

The Rwandan government has adopted peer education as a strategy to prevent HIV infection among in-school youth [5,6]. Peer education is “the process whereby well-trained and motivated young people undertake informal or organized educational activities with their peers (those similar to themselves in age, background or interests) over a period of time, aimed at developing their knowledge, attitudes, beliefs and skills and enabling them to be responsible for and protect their own health” [7]. Since 1998, the Rwandan Government has been installing anti-AIDS clubs in secondary schools. Ten years later 98% of secondary schools had installed such clubs. However, the anti-AIDS clubs often remain inactive due to a lack of guidance, financial and material support [8].

Over the past few years the Rwandan Government and several organizations, including Voluntary Service Overseas, Population Services International, Africa Humanitarian Action, Human Development Initiative and the Rwandan Red Cross Society, have selected a number of schools to provide support for the anti-AIDS clubs. In most cases, this support is temporary and project-based, and consists of training a number of selected students to become a peer educator in their school. The effectiveness of these activities has not yet been thoroughly evaluated. Since this method of HIV prevention will remain important in Rwanda for the next few years, and it is a common practice in other African countries, we found it important to evaluate its effectiveness and, if needed, to formulate recommendations. This paper evaluates the effectiveness of the HIV prevention peer education intervention implemented by the Rwandan Red Cross Society in the district of Bugesera in increasing HIV related knowledge, reducing sexual risk behaviors and changing attitudes. Hence this paper adds to the knowledge base of the effectiveness of HIV prevention interventions for young people in sub-Saharan Africa, and of peer education in particular.

## Methods

### The peer education intervention

This study assesses the effectiveness of the peer education program on young people’s HIV knowledge, attitudes and self-reported sexual behavior. The intervention was based on an integrated theoretical framework that included aspects of the Theory of Reasoned Action, the Social Learning Theory, the Diffusion of Innovations Theory, and the Health Belief Model. The intervention delivery was based on participatory learning techniques. The intervention development was the result of a collaboration between the Rwandan Red Cross and two expert organizations in the field of HIV and sexual and reproductive health, and was based on a number of peer education manuals [7,9,10].

The intervention took place in all fifteen secondary schools in the district of Bugesera (Rwanda) and was developed and implemented by the Rwandan Red Cross. The general objective of the peer education program was to reduce sexual risk behavior and to promote sexual and reproductive health in the secondary school communities by activating the anti-AIDS-clubs in the schools. The design of the program was informed by experiences with peer education in a different district in Rwanda, several manuals on effective peer education [10,11] and the expertise of two organizations specialized in sexual health. The intervention consisted of an initial six-day training for five students (peer educators) of each participating school, as well as for one teacher per school who was tasked with supporting the peer educators in their daily activities. The training consisted of information on the Red Cross and its main principles, HIV/AIDS, sexually transmitted diseases, family planning and pregnancies, the role of the peer educator (what is expected of a peer educator and what is the deontology of a peer educator?) and teaching methods (how to best approach students and how to transmit messages and counsel?). School principals attended a half-day information session on the program. The peer educators were selected by the disciplinary teacher, who lives in the school and knows the students well, based on a number of predefined criteria (personal characteristics, sex, study year).

The training for the peer educators was organized in July 2009 and intervention activities in the schools started in August 2009. Additional trainings were planned in the second part of the intervention, but did not take place (see Discussion section). In September 2009 a large event uniting all intervention schools was organized to launch the intervention. During the course of the intervention, the peer educators were tasked with teaching their fellow students how to adopt positive and responsible behaviors, such as respect within relationships and personal responsibility for protective behavior. This was done through group and individual counseling, drama performances, songs and other interactive methods. Throughout the duration of the intervention, the activities of the peer educators were monitored and followed-up by the district Red Cross coordinator. Peer educators had to hand in an activity report each trimester. The intervention ended in November 2010. The implementation process and a process analysis is described in detail elsewhere [12].

### Study design and sample size

The study is a non-randomized controlled trial including eight intervention and six control schools. The study assessed knowledge, attitudes and behaviors of the students three times over a period of eighteen months: March 2009 (Baseline), March 2010 (T1) and September 2010 (T2).

We based the sample size calculation on the study objective of assessing whether or not the intervention influenced the time trend in condom use and recent history of sexual intercourse. Sample size calculations were conducted with Wald tests for the odds ratio resulting from regression models with two binary variables (intervention/control and T0/T1 or T0/T2) and their interaction. For logistic regression models, a minimum of 1,241 observations are required to detect an adjusted odds ratio of 2 or more with 80% power at the 0.05 significance level, under conservative assumptions of 30% baseline prevalence of the outcome variable and no changes over time in the control group [13]. For linear regression models, a minimum of 348 observations are required to detect a small standardized effect size (Cohen’s d) of 0.3 with 80% power at the 0.05 significance level [14,15]. Further, we assumed a design effect of 2, due to possibly strong correlation of repeated measurements from the same participant (T0/T1/T2), resulting in a minimum of 2,482 observations required from 1,241 participants. Anticipating a 25% loss to follow-up, we increased the target sample size to 1,655 participants at T0.

### School and participant selection

Schools were selected on a purposive basis. We aimed to include the greatest variety of schools in the study and applied several selection criteria: education offered (lower/higher secondary education), location (urban/rural), religious background, number of students (small/large) and funding (public/private). All selected schools were willing to participate in the study. Since no roads directly connect intervention and control sites, cross-site contamination was unlikely.

The study targeted all students who were in their second and fifth year, since they had a higher chance of still being in school at the end of the survey. Drop-out rates are highest after the third year (the end of lower secondary education) and of course the sixth and final year. A coding system guaranteeing confidentiality, separately storing identifying information and questionnaire answers, was used to match students over the three waves. At T1 and T2 schools were visited up to three times to retrieve students. In the schools included in our study, no other organizations were implementing activities in the field of HIV/AIDS or sexual and reproductive health (Table 1).

**Table 1:** Characteristics of participating schools

**School**	**Location I = intervention C = control**	**Location (urban/rural)**	**Lower/higher secondary education**	**Number of students (2009)**	**Public or private**	**Religious background**
School 1	Bugesera (I)	Urban	lower + higher	>750	private	mixed
School 2	Bugesera (I)	Rural	lower + higher	>750	private	Catholic
School 3	Bugesera (I)	Rural	lower	251-500	public	mixed
School 4	Bugesera (I)	Rural	lower	251-500	public	mixed
School 5	Bugesera (I)	Urban	lower	501-750	public	mixed
School 6	Bugesera (I)	Rural	lower + higher	<250	private	Islamic
School 7	Bugesera (I)	Urban	higher	501-750	public	mixed
School 8	Bugesera (I)	Rural	lower + higher	>750	public	mixed
School 9	Rwamagana (C)	Rural	lower + higher	501-750	public	mixed
School 10	Rwamagana (C)	Rural	lower + higher	501-750	public	mixed
School 11	Rwamagana (C)	Rural	lower + higher	251-500	public	mixed
School 12	Rwamagana (C)	Urban	lower	<250	private	Catholic
School 13	Rwamagana (C)	Urban	lower	<250	private	mixed
School 14	Rwamagana (C)	Urban	lower + higher	>750	private	Catholic

### Procedure

The questionnaire was developed in French, translated in Kinyarwanda and back translated in French. It was tested for comprehensibility on a population of 30 students in the first year of secondary education. The questionnaires were self-administered in classrooms or refectories. Data-entry was done electronically using Optical Mark Recognition software.

Before the start of the survey, the students gave written informed consent after concepts of voluntary participation and confidentiality were explained to them. School principals signed an informed consent form agreeing that their school would be used as a study site and that students would be requested to complete questionnaires. Parental consent was waived in the ethical review process, based on two arguments. First, practical considerations: parents live far from the schools and visit rarely, no full address details or phone numbers were available, and illiteracy is high. Second, we argued for a developmental approach to adolescence and adulthood and stressed the importance of collecting data directly from adolescents. Based on several guidelines [16-18] and scientific literature [19-21] we argued that adolescents have the cognitive capacity to take decision concerning participation in social and human science research.

### Statistical analysis

Statistical analyses were performed using Stata version 11 (Stata Corporation, College Station, TX) and SAS version 9.2 (SAS Institute Inc., Cary, North Carolina). Since the allocation of schools to the intervention and control group was not randomized, we initially evaluated eight variables (described further) to see if there were significant differences between baseline values in the intervention and control groups (see Table 2). Then we calculated propensity scores and participants with propensity scores outside of the area of common support ([0.17, 0.98]) were excluded from subsequent analyses [22]. Propensity scores are used to reduce selection bias when assignment to study arms is not randomized. A participant’s propensity score is its probability of being assigned to a specific study arm given a set of known covariates [23].

**Table 2:** Socio-demographic characteristics of respondents at baseline before and after adjusting for propensity score

**Variable**	**Intervention allocation**		**P-Value**	**P-Value**
	**Control group**	**Intervention group**		
	**Rwamagana**	**Bugesera**	Before Adjustment*	After Adjustment**
**Sex n (%)**				
Male	417 (47.55)	543 (50.70)	0.17	0.80
**Age**				
Mean (sd)	17.60 (2.30)	18.41 (2.18)	<0.01	0.95
**Ever had sex n (%)**				
Yes	196 (22.55)	302 (28.76)	<0.01	0.91
**Condom at last intercourse n (%)**				
Yes	52 (6.39)	99 (10.25)	0.13	0.98
Never had sex	679 (83.42)	755 (78.16)	0.21	0.94
**Intercourse in last 6 months n (%)**				
Yes	62 (7.09)	118 (11.24)	<0.01	0.98
**Ever tested for HIV n (%)**				
Yes	369 (43.11)	564 (56.29)	<0.01	0.73
Don’t know	17 (1.99)	19 (1.90)	0.51	0.88
**Socio-economic status n (%)**				
Middle	399 (45.65)	349 (32.80)	<0.01	0.63
High	47 (5.38)	38 (3.57)	<0.01	0.11
**Live during the year n (%)**				
Boarding school, off campus	14 (1.64)	209 (20.47)	<0.01	<0.05
Parents/Family	283 (33.06)	430 (42.12)	<0.01	0.90
**Baseline HIV knowledge**				
Mean (sd)	6.12 (1.80)	5.91 (1.90)	<0.05	0.94

*Calculated using simple regression models with the baseline characteristic as the outcome of interest, and the intervention group as the independent variable.

**Calculated using multiple regression models. Observations whose propensity scores fell outside of the minimum and maximum values for the opposite group were eliminated from the analysis. A model was fit with the baseline characteristic as the outcome of interest, and the intervention group as the independent variable. It was adjusted for the propensity score.

Marginal linear and logistic regression analyses, using Generalized Estimating Equations (GEE), were conducted to determine the likelihood of experiencing different outcomes based on which group the participant belonged to, while accommodating for repeated, correlated measures. Specifically and most importantly for this analysis, these models allow for investigation of group effects, time effects, and group by time interactions. Our analysis specified an unstructured correlation matrix and a binomial or Gaussian distribution depending on which dependent variable was analysed. Correlation between students within schools was ignored in the analysis, as it was weak and non-significant (p > 0.05).

### Variables

The independent variable in this study was exposure to the peer education program, operationally defined as attending a school where the peer education intervention was deployed.

Degree of participation in the program was operationalized using a scale made up of six variables measuring participation in six program activities (e.g. drama plays or small-group counseling). For each activity the respondent could indicate if he/she did not participate, participated passively (observed) or actively participated in the activity. The participation scale, including all six activities, ranges from 0 (no participation at all) to 12 (very active participation) (Cronbach’s alpha: 0.834).

The study evaluated seven dependent variables that assessed a student’s knowledge of HIV protection modes, attitudes toward HIV/AIDS, and sexual behavior. Knowledge of HIV protection modes was measured with 11 items proposing true and false statements about ways of protection, which were consequently summed to get a score from 0 (all answers incorrect) to 11 (all answers correct). Attitudes towards HIV/AIDS were divided into three attitudinal constructs. Perceived susceptibility to HIV (range 0-16, Cronbach’s alpha 0.67) was measured using the validated 4-items scales of Lux & Petosa [24]. Perceived severity of HIV was measured in one single item ‘At present, the danger of AIDS has almost passed’ (agree/disagree). Enacted stigma was measured by two items asking if the students would refuse to be taught by a HIV-positive teacher and if they thought HIV-positive students should be expelled from school (scale 0-1, Cronbach’s alpha 0.76). Sexual behavior was measured through three variables: did you ever have sexual intercourse? (no/yes); did you have sex in the last 6 months? (no/yes); did you use a condom at last sexual intercourse? (no/yes).

The propensity score included eight socio-demographic and behavioral baseline variables, chosen because they demonstrated significant differences between intervention and control students: 1) sex: boy/girl; 2) age; 3) ever had sex: yes/no; 4) condom use at last intercourse: yes/no/don’t remember; 5) had sex in the last six months: yes/no; 6) having been tested for HIV: yes/no/don’t know; 7) socio-economic status: a constructed variable, made up of five questions on possessions of the respondent’s family and subsequently categorized into low, middle, or high socio-economic status; 8) living situation during the school year: boarding school on school ground/boarding school outside school grounds/with parents or family.

Two additional candidate confounding variables were evaluated in the models, because of their potential impact on sexual behavior: 1) alcohol use (never/once a week or more/once a months or more) since sexual risk behavior has often been associated with alcohol use [25-27]; 2) sexual self-concept using an adapted scale made up of 13 4-point Likert items, with scores ranging from 13 to 52 (Cronbach’s alpha of 0.60) [28]. Sexual self-concept has been studied and found to be of influence on sexual behavior and sexual decision making [29-31].

### Ethical approval

The study was approved by the Ethics Commission of the Ghent University Hospital (2008/485), the Rwanda National Ethical Committee (42/RNEC/2009), the Rwandan Institute for Statistics (130/2009/INSR) and the Rwandan National AIDS Control Commission (0135/CNLS/2009/S.E).

## Results

The total retention rate after the third survey at 18 months was 71.8% (1400/1950). Reasons for not completing the study were drop-out of school (63%), illness (9%) and absence from school at the time of survey (28%). The retention rate was higher in the control group: 65.1% versus 79.7% (p < 0.001). Table 2 shows the socio-demographic characteristics and baseline sexual behavior of respondents. On a number of factors there were significant differences between the intervention and control groups at baseline. Although the median age was the same, the average age of students was higher in the intervention group than in the control group. There are more intervention students following technical education and less in accounting. Control students have a higher socio-economic status. As for their sexual behavior, more intervention students were sexually active, but they did not have more sex in the 6 months preceding the survey. More intervention students had been tested for HIV, while control students had more knowledge of HIV. After adjustment for propensity score, only the living situation was significantly different for intervention and control students. Of the initial 1950 students, 1588 were retained in the main analysis after dropping participants who were not in the area of common support from the propensity score analysis (n = 362).

Table 3 and 4 show the evolution in key outcome variables over time for the intervention and control group. Over the period of the intervention, the number of sexually active students increased, the proportion of students who had sex in the six months preceding the survey increased and condom use at last sex increased. These evolutions were not significantly different in the intervention and control group.

**Table 3:** Sexual behavior, knowledge and attitudes of respondents at Baseline, T1 and T2, by study setting

	**Intervention group**			**Control group**		
	**Baseline**	**T1**	**T2**	**Baseline**	**T1**	**T2**
**ever had sexual intercourse** n (%)	186 (22.01)	192 (30.87)	252 (42.93)	124 (16.69)	166 (26.35)	203 (32.17)
**had sex in last 6 months** n (%)	93 (11.01)	75 (12.40)	85 (14.46)	51 (6.86)	58 (9.24)	49 (7.84)
**used condom at last sex (of those sexually active)** n (%)	83 (44.62)	55 (40.74)	63 (47.37)	47 (37.90)	45 (41.28)	44 (40.74)
**knowledge of HIV protection modes** mean (median)	7.15 (8)	7.24 (8)	9.36 (10)	7.23 (8)	7.49 (8)	9.64 (10)
**perceived susceptibility to HIV** mean (median)	7.32 (7)	6.81 (7)	7.03 (7)	6.79 (7)	6.55 (6)	6.45 (6)
**perceived severity of HIV** n(%) (high)	203 (27.77)	129 (22.05)	133 (23.79)	145 (20.71)	127 (20.65)	94 (15.36)
**enacted stigma** mean (median)	0.35 (0)	0.19 (0)	0.24 (0)	0.18 (0)	0.10 (0)	0.12 (0)

**Table 4:** Results of the Peer Education intervention on sexual behavior, HIV knowledge and attitudes

	**Ever had sexual intercourse**	**Had sex in last 6 months**	**Condom use at last sex**	**Perceived Severity**	**Knowledge of HIV protection modes**	**Perceived susceptibility**	**Enacted stigma**
	**OR [95%CI]**	**OR [95%CI]**	**OR [95%CI]**	**OR [95%CI]**	**Bèta-coefficient [95%CI]**	**Bèta-coefficient [95%CI]**	**Bèta-coefficient [95%CI]**
Intervention (ref. control group)	1.16 [0.85-1.57]	1.28 [0.82-1.98]	1.22 [0.76-1.94]	1.41 [1.09-1.83]*	−0.11 [−0.23-0.00]	0.57 [0.28-0.86]*	0.18 [0.12-0.24]*
Month (T1) (ref. baseline)	1.84 [1.58-2.15]*	1.48 [1.05-2.09]*	1.28 [0.73-2.26]	0.98 [0.78-1.23]	0.25 [0.16-0.35]*	−0.22 [−0.45-0.01]	−0.08 [−0.13- -0.04]*
Month (T2) (ref. baseline)	2.38 [2.00-2.83]*	1.20 [0.81-1.78]	1.31 [0.74-2.32]	0.71 [0.56-0.91]*	2.40 [2.29-2.51]*	−0.32 [−0.55- -0.09]	−0.07 [−0.11- -0.03]*
Intervention* Month T1	0.97 [0.79-1.20]	0.80 [0.50-1.29]	0.73 [0.35-1.53]	0.74 [0.54-1.02]	−0.15 [−0.30- -0.01]*	−0.28 [−0.64-0.08]	−0.08 [−0.15- -0.01]*
Intervention* Month T2	1.29 [1.00-1.67]	1.18 [0.69-2.01]	0.75 [0.36-1.57]	1.12 [0.80-1.57]	−0.19 [−0.36- -0.02]*	−0.00 [−0.36-0.35]	−0.05 [−0.12-0.03]
Alcohol – at least once a month (ref. never)	2.16 [1.65-2.83]*	1.69 [1.23-2.32]*	—	—	—	—	—
Alcohol – at least once a week (ref. never)	3.97 [2.65-5.95]*	4.66 [3.12-6.97]*	—	—	-—	-—	—-
Sexual Self-Concept	1.06 [1.04-1.08]*	1.04 [1.02-1.06]*	—	—	—	−0.05 [−0.05- -0.03]	−0.01 [−0.01- -0.00]*

*significant at level 0.05.

All models controlled for the propensity score variable.

We found an increase in knowledge both in the intervention and control students, especially in the second part of the intervention. This increase was significantly slower in the intervention group, although in absolute numbers the difference was not very large. Reported enacted stigma was high at baseline, especially in the intervention group. The data showed that the intervention significantly reduced enacted stigma, especially in the first part of the intervention, but the trend remained visible at T2. No significant results were found for perceived susceptibility to or perceived severity of HIV.

Alcohol use (both occasional and frequent) had a significant impact on being sexually active and on recent sexual activity. Respondents with a high sexual self-concept were more likely to be sexually active and to have had sex in the last six months, and less likely to report enacted stigma.

**Figure 1:**
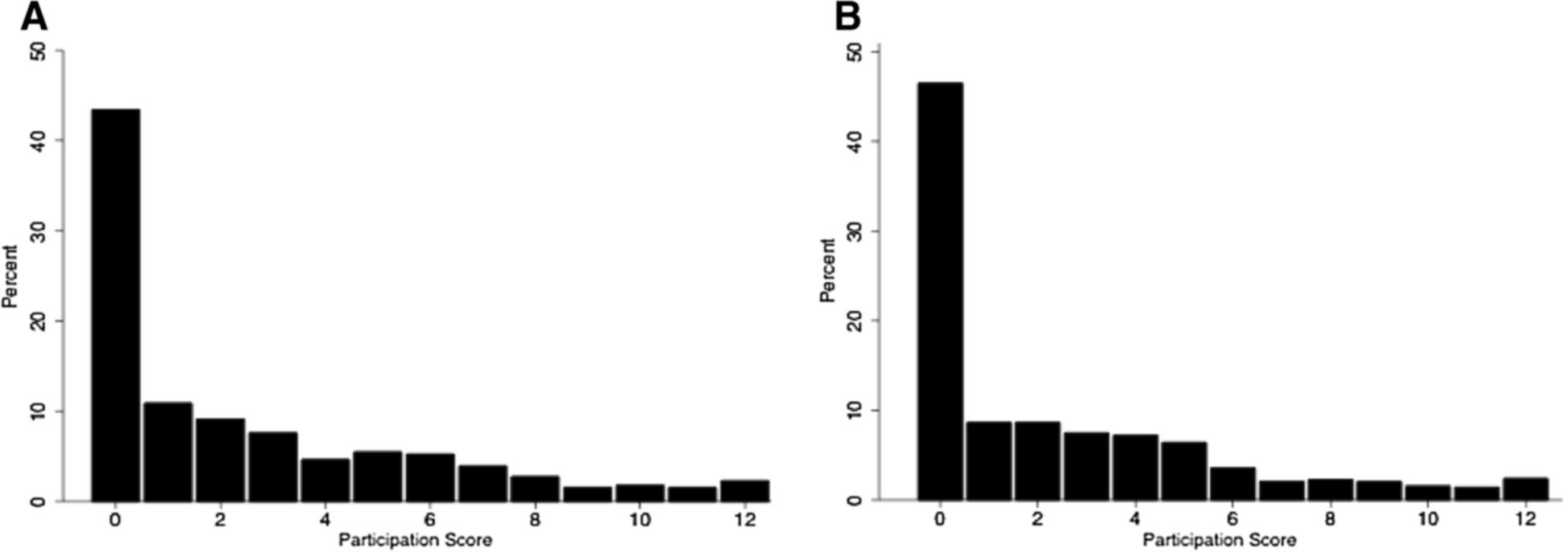
Distribution of participation scores at T1 (A) and T2 (B).

Figure 1 shows that a large proportion of students in the intervention schools did not participate at either point in time (43.4% at T1 and 46.5% at T2) and only 13.7% and 11.6% of students had a participation score above six at T1 and T2 respectively. In a supplementary dose–response analysis among students from the intervention schools only, we investigated whether more intensive participation in the peer education program affected any of the seven dependent variables. Only for knowledge of HIV we observed a statistically significant time*participation score at T2, yet the effects of higher participation on were minimal and not behaviorally meaningful.

## Discussion

### Limited effectiveness of the peer education intervention

We observed limited effectiveness of the peer education intervention in increasing knowledge, changing attitudes and reducing sexual risk behavior. The intervention did not seem to effectively alter sexual risk behavior. Knowledge of HIV protection modes did increase somewhat, but the increase was actually larger in the control group. We could not find a sound explanation for this observation. On the other hand, the intervention did significantly reduce enacted stigma. This could indicate that the peer education program succeeded in creating a more positive, less stigmatizing climate, pulling it out of the taboo sphere.

The results are comparable to other evaluation studies of peer education interventions for youth. Individual and review studies have shown that peer education interventions do not completely succeed in their main objective, i.e. reducing sexual risk behavior [32-37]. The most recent review on the effectiveness of peer education HIV prevention interventions [38] identified four evaluations of peer education interventions in sub-Saharan Africa that reported on self-reported behavior using a quasi-experimental design [39-42], not showing compelling evidence that peer education works for young people in this context. A literature study on the effectiveness of all types of HIV prevention interventions for young people in sub-Saharan Africa done in 2006 and recently updated indicated weak evidence that peer-led interventions are effective and recommended against scale-up [43,44].

### Study limitations

We are aware of several limitations of this study. First, since the intervention was planned to be implemented in all schools in one district in Rwanda it was not possible to randomize schools for participation in the intervention. Moreover, the intervention district was chosen because of its lower socio-economic status and its need for the intervention. Unsurprisingly, we found socio-demographic and behavioral differences between intervention and control students at baseline. To counter these differences, we adjusted for propensity scores in all analyses. Second, all schools, both in the intervention and control site, were asked if other HIV prevention interventions were taking place in the schools. Even if this was not the case, it cannot be excluded that regional or national prevention campaigns intervened with the interventions, that interventions with lingering effects had been implemented prior to the evaluation, or that in some schools teachers might be more actively involved in spreading HIV prevention messages, e.g. in biology classes. Third, we operated from the assumption that the messages of the intervention would reach, one way or another, all students in the schools. The interventions were freely accessible for all students of the intervention schools. However our analysis showed that a large number of students did not participate in the intervention at all, while only a few participated very actively. Nevertheless, differences in outcomes among students with low and high participation levels were negligibly small. Finally, even though we have regular reports of peer educators’ activities and paid several visits to the schools, we were not present during all the activities and therefore cannot ascertain the quality of all activities in each school.

### Reasons for observed limited effectiveness

Based on our evaluation study and existing literature we identified several factors that can help to explain the limited impact of this intervention, in particular, and of peer education for young people in general. These factors are associated with: 1) the implementation of the intervention; 2) the design of the intervention; 3) the underlying assumptions of peer education for young people; 4) appropriate indicators of sexual behavior of young people.

### Factors associated with the implementation of the intervention

The intervention studied in this paper was limited by implementation issues, partly explaining the lack of effectiveness. During the second half of the intervention internal problems arose in the organization, leading to limited monitoring and follow-up of the peer educators, and failure to provide the second round of training for the peer educators [12]. Consequently, in the second part of the intervention, we observed a reduction in the number of activities organized by the peer educators in all schools. In the activity reports of the second part of the intervention, more peer educators requested additional support of the intervention coordinator. However, if this was the only reason for the lack of effectiveness, we would have seen better results after the first part of the intervention.

### Factors associated with the design of the intervention

Notwithstanding that the intervention discussed in this paper was thoroughly developed, based on previous experience, peer education manuals and with the input from expert organizations, there were some lacunas in the intervention design.

The objectives of the intervention are very broad (to reduce sexual risk behavior and to promote sexual and reproductive health in the secondary school communities), as is the case in many other peer education interventions, e.g. [45-49], while the methodologies used are rather limited (informative, sensitizing methodologies such as theatre, songs, counseling). It has been amply demonstrated and discussed that increasing knowledge alone will not change sexual behavior [50-52], since sexual behavior is also determined by a number of other factors. For example, we cannot expect young people to use more condoms by only talking about condoms and not providing them in the schools.

Furthermore, the intervention focuses on the individual, while sexual behavior is influenced by a large number of factors on different levels: personal, inter-personal, institutional, socio-cultural, structural (e.g. the socio-ecological model of Bronfenbrenner [53]). Behavior change can only be reached by tackling all these levels. Mason-Jones [35] explained after evaluating the lack of effectiveness of a peer education program: “It may be that social factors are so influential that an individualized health education program cannot hope to make changes”.

It is our conviction that we set the expectations of peer education interventions too high. It would be more realistic to recognize these interventions in their true value in contributing to a more positive, less stigmatizing climate, and to complement them with other types of interventions, such as youth-friendly services, condom distribution, community involvement and structural approaches.

### Factors associated with peer education as a prevention strategy

Since many Rwandan school-going youth stay in a boarding school and only return to their families two or three times a year, they have no other option than to rely on peers or teachers for HIV/SRH information. However, this does not mean they *want* to rely on them. In our study students were asked to indicate the two main channels through which they would prefer to receive information on HIV: friends ranked sixth as a preferred source of information, after radio, parents, television, teachers and medical experts (docters/nurses).

This finding is supported by studies from other countries. Young people in Uganda prefer receiving HIV information from formal sources. They rank friends last and mass media and teachers first as preferred prevention sources [54]. In Cameroon a study among urban youth shows that only 3% of respondents named their friends as people whose opinion they value, while 93% mentioned family members [55]. A study among Canadian youth demonstrated that, although they indicate friends as their main source, young people prefer receiving sexual health information from professionals [56]. A study from the United Kingdom stressed the important role of parents in sex education, and showed that young people prefer to be taught about sexual health by health professionals [57].

Peer education implies that certain members of a group (peer educators) can be influential in convincing their peers to change their behavior. The strategy has proven successful in other fields of health promotion (e.g. [58,59]). However, when it comes to HIV prevention among young people, not disregarding the capacities they have, it is a very tall order to expect a young person – possibly discovering his/her sexuality him/herself - to act as an expert and guide, counsel, teach and advise peers on a personal, sensitive and complex issue as sexuality. Furthermore, when it comes to young people, the notion of ‘peer’ oftentimes refers to someone of the same age. This is a very simplistic notion: even though they might be of approximately the same age, this does not mean they have a similar background, similar experiences, similar values and norms [60]. Besides personal characteristics, a peer educator’s credibility is determined by their own behavior and by how they transmit messages. A study of a peer education drug prevention intervention found that young people value experience-based and message-based credibility more than the peer educators’ personal characteristic ( [61] in [60]).

### Factors associated with the evaluation of the intervention

The ultimate goal of HIV prevention interventions is a reduced incidence of HIV in young people. While directly measuring HIV incidence is often not possible, the envisaged intervention effect is operationalized by measuring self-reported sexual behavior. To this end, in this and many other evaluation studies, internationally recognized indicators are used: ‘condom use at last sex’ (if this increases the intervention is considered successful), ‘recent sexual activity’ (if this decreases, the intervention is considered successful), the ‘number of sexual partners in the last 6 months’ (if this decreases the intervention is considered successful). We argue that these indicators might not be adequate to measure the actual risks taken by the respondents. By using these indicators individually and by neglecting the relationships or context in which these sexual activities take place, these indicators ignore that young people can have healthy sexual relationships. For example, the indicator ‘condom use at last sex’ might hide an increase in young people that are in a monogamous relationship, and decide not to use a condom after a negative HIV test. Or why would having a large number of sexual partners be negative for one’s sexual health, if the sexual intercourse is consensual and protected? An indicator that would appropriately measure sexual risk behavior should include aspects of exposure (relationship and partner characteristics), transmission (type of sex and protective measures), and preferably also infectiousness (HIV infection and stage of infection of the partners). The development of such contextualized, composite sexual behavioral measures is essential to measure the real risks young people are taking, hence to determine the real effectiveness of HIV prevention interventions. Our intervention did control for individual characteristics influencing sexual behavior (alcohol use and sexual self-concept), but did not control for relational and contextual characteristics.

## Conclusions

### Recommendations for future interventions and evaluation studies

Peer education is an attractive tool for HIV prevention because it makes use of existing social processes and actively involves young people in the intervention. However, given the observed limited effectiveness, it might be necessary to re-evaluate the role of peer educators. First, program planners must set realistic expectations for peer education. Peer education based on information sharing only, will never on its own change sexual behavior to a sufficiently large extent. It can however be valuable in creating a less-stigmatizing climate around sexuality and in breaking taboos. Second, while peer educators are now the centre of the intervention, informing - counseling and advising fellow students - this role needs to be redefined. Peer educators could be deployed as focal points: beside spreading information on HIV through theatre, songs and discussions, they should be the ones who are aware of key specialists and services to which they can refer. Peer education should not be a stand-alone intervention and should be embedded in a larger strategy. Third, peer education interventions seemingly actively involve young people in the prevention efforts. However, true participation goes further than only involving them in the implementation phase. Young people can participate and provide their input in the needs assessment, intervention development, the follow-up and in monitoring and evaluation. Their input in these phases might be more valuable than in the implementation of the intervention.

Evaluations of HIV prevention interventions need to be more aware of contextualizing outcome measures: condom use and sexual activity as such are not entirely adequate indicators of sexual risk behavior, but need to be complimented by additional indicators related to the nature and context of sexual relationships.

Finally, we must be clear about what we really want to accomplish with HIV prevention interventions. Focusing narrowly on ‘sexual activity’ and ‘condom use’ is not optimal, since these behaviors are the result of a decision process influenced by many factors that are inadequately addressed by current peer education interventions. In our view, it would be more useful to focus on the decision-making process itself and empower young people to make their own conscious, responsible decisions. A useful concept in this perspective is ‘sexual competence’ [62], meaning that, sexual intercourse should be protected, consensual, undertaken without regret and as a result of an autonomous decision.

## Competing interests

The authors declare that they have no competing interests.

## Authors' contributions

KM initiated the study, developed the protocol, collected the data, participated in the analysis and took the lead in writing the paper. RB was responsible for data analysis and writing. WD was responsible for data analysis and writing. KR contributed to data collection and writing. RVR contributed to the set-up of the study and provided support in data collection, analysis and writing. MT contributed to the set-up of the study and provided support in data collection and writing. All authors read and approved the final manuscript.

## Pre-publication history

The pre-publication history for this paper can be accessed here:

http://www.biomedcentral.com/1471-2458/12/729/prepub
